# Dissociation of mitochondrial depolarization from cytochrome *c* release during apoptosis induced by photodynamic therapy

**DOI:** 10.1054/bjoc.2000.1714

**Published:** 2001-04

**Authors:** S-M Chiu, N L Oleinick

**Affiliations:** Department of Radiation Oncology and The CWRU/Ireland Comprehensive Cancer Center, School of Medicine, Case Western Reserve University, Cleveland, OH, 44106, USA

**Keywords:** photodynamic therapy, phthalocyanine Pc 4, mitochondria, apoptosis, membrane potential, cytochrome *c*

## Abstract

Photodynamic therapy (PDT) with the phthalocyanine photosensitizer Pc 4 induces rapid apoptosis in mouse lymphoma (LY-R) cells, initiating with the release of cytochrome *c* from mitochondria. It has been proposed that the opening of the mitochondrial membrane permeability transition pores, which results in the dissipation of the mitochondrial membrane potential (Δψ_m_), is essential for the escape of cytochrome *c* from mitochondria into the cytosol as well as for apoptotic cell death. Therefore, we have assessed the correlation between the loss of Δψ_m_ and the release of cytochrome *c* following PDT. Treatment of LY-R cells with 300 nM Pc 4 and 60, 90 or 120 mJ/cm^2^of red light resulted in apoptosis of 80–90% of the cells, accompanied by >20-fold elevation in caspase-3-like activity within one h. At all 3 doses of PDT employed here, the majority of the cytochrome *c* was released from mitochondria at 15 min after irradiation, as determined by an immunohistochemical method. In contrast, the loss of Δψ_m_ following PDT, as monitored by the uptake of JC-1 or Rh-123, depended on the PDT dose and the post-treatment time. In spite of the release of cytochrome *c* at 15 min after each of the 3 doses, a corresponding loss of Δψ_m_ was observed only for those cells that received the highest dose of PDT. Virtually all cells that received one of the lower doses of PDT (300 nM Pc 4 plus 60 or 90 mJ/cm^2^) maintained normal Δψ_m_. Hence, our results support the conclusion that the release of cytochrome *c* from mitochondria resulting from Pc 4-PDT-induced photodamage is independent of the loss of Δψ_m_. Therefore, it is important to consider a range of doses of this or other apoptotic stimuli in deciphering the relationship of metabolic responses that contribute to apoptosis. © 2001 Cancer Research Campaign http://www.bjcancer.com


*British Journal of Cancer* (2001) **84**(8), 1099–1106
© 2001 Cancer Research Campaign
doi: 10.1054/ bjoc.2001.1714, available online at http://www.idealibrary.com on IDEAL®

### Dissociation of mitochondrial depolarization from cytochrome *c* release during apoptosis induced by photodynamic therapy

**S-M Chiu and NL Oleinick**

Department of Radiation Oncology and The CWRU/Ireland Comprehensive Cancer Center, School of Medicine, Case Western Reserve University, Cleveland, OH 44106, USA

**Summary** Photodynamic therapy (PDT) with the phthalocyanine photosensitizer Pc 4 induces rapid apoptosis in mouse lymphoma (LY-R) cells, initiating with the release of cytochrome *c* from mitochondria. It has been proposed that the opening of the mitochondrial membrane permeability transition pores, which results in the dissipation of the mitochondrial membrane potential ($\Delta\psi_m$), is essential for the escape of cytochrome *c* from mitochondria into the cytosol as well as for apoptotic cell death. Therefore, we have assessed the correlation between the loss of $\Delta\psi_m$ and the release of cytochrome *c* following PDT. Treatment of LY-R cells with 300 nM Pc 4 and 60, 90 or 120 mJ/cm$^2$ of red light resulted in apoptosis of 80–90% of the cells, accompanied by >20-fold elevation in caspase-3-like activity within one h. At all 3 doses of PDT employed here, the majority of the cytochrome *c* was released from mitochondria at 15 min after irradiation, as determined by an immunohistochemical method. In contrast, the loss of $\Delta\psi_m$ following PDT, as monitored by the uptake of JC-1 or Rh-123, depended on the PDT dose and the post-treatment time. In spite of the release of cytochrome *c* at 15 min after each of the 3 doses, a corresponding loss of $\Delta\psi_m$ was observed only for those cells that received the highest dose of PDT. Virtually all cells that received one of the lower doses of PDT (300 nM Pc 4 plus 60 or 90 mJ/cm$^2$) maintained normal $\Delta\psi_m$. Hence, our results support the conclusion that the release of cytochrome *c* from mitochondria resulting from Pc 4-PDT-induced photodamage is independent of the loss of $\Delta\psi_m$. Therefore, it is important to consider a range of doses of this or other apoptotic stimuli in deciphering the relationship of metabolic responses that contribute to apoptosis. © 2001 Cancer Research Campaign http://www.bjcancer.com

**Keywords**: photodynamic therapy; phthalocyanine Pc 4; mitochondria; apoptosis; membrane potential; cytochrome *c*

The mitochondrion plays a central role in the control of apoptosis (Kroemer et al, 1997; Green and Reed, 1998) by releasing the apoptogenic proteins apoptosis-inducing factor (AIF) (Susin et al, 1999) and cytochrome *c* into the cytosol. The released proteins activate pathways essential for carrying out the morphological and biochemical changes initiated by a variety of death stimuli (Gross et al, 1999). For example, cytochrome *c*, which normally resides between the mitochondrial outer and inner membranes and serves as a diffusible electron carrier in the intermembrane space (Cortese and Hackenbrock, 1993), has been demonstrated to be a co-activator of caspase-9, which in turn activates caspase-3 (Liu et al, 1996; Kluck et al, 1997; Yang et al, 1997). The active caspase-3 then activates CAD (caspase-activated DNase) through proteolytic inactivation of its bound inhibitor ICAD (Liu et al, 1997; Enari et al, 1998). Active CAD translocates from the cytosol into the nucleus to initiate chromatin condensation and DNA fragmentation.

Although the mechanism by which the apoptosis-inducing proteins are released from mitochondria remains unclear, the release is regulated by members of the Bcl-2 family of proteins in that anti-apoptotic members, such as Bcl-2 and Bcl-xL, suppress, and pro-apoptotic members, such as Bax and Bak, promote the release (Reed et al, 1998; Gross et al, 1999). The Bcl-2 family proteins either reside on the mitochondrial membrane or are translocated there during apoptosis. Several models have been proposed for the release of cytochrome *c* from mitochondria (Martinou et al, 2000). One model proposes that the release depends on the mitochondrial permeability transition (PT) (Susin et al, 1998), which represents an abrupt increase in the permeability of the mitochondrial inner membrane to molecules of less than 1500 Da (Zoratti and Szabo, 1995; Lemasters et al, 1998). It is believed that PT is a consequence of the opening of protein channels or pores in the membrane, called permeability transition pores (PTP) (Kroemer et al, 1997; Green and Reed, 1998). The ill-defined PTP is located at sites of contact of the inner and outer membranes and consists of several protein components, including the adenine nucleotide translocator (ANT) and the voltage-dependent anion channel (VDAC). According to this model, the opening of the PTP would lead to the influx of water and solutes into the matrix, causing swelling of the mitochondria, and eventually the rupture of the outer membrane (Vander Heiden et al, 1997). The model predicts that opening of the PTP would result in dissipation of the electrochemical gradient across the inner membrane ($\Delta\psi_m$), resulting in mitochondrial depolarization and disruption of respiration.

However, evidence that runs contrary to this model has been reported. First, time-course studies have demonstrated that release of cytochrome *c* can precede membrane depolarization (Yang et al, 1997; Bossy-Wetzel et al, 1998). And second, the release of cytochrome *c* can occur in the absence of mitochondrial depolarization (Kluck et al, 1997; Eskes et al, 1998; Finucane et al, 1999a, 1999b; Goldstein et al, 2000). Alternative models (Martinou et al,

*Received 11 September 2000*
*Revised 13 December 2000*
*Accepted 15 December 2000*

*Correspondence to:* NL Oleinick

2000) have therefore been proposed that depend on the creation of pores or channels in the outer membrane large enough for the passage of cytochrome *c*. Pro-apoptotic Bcl-2 family proteins, such as Bax, may participate in the channel formation.

In this study, we investigated the role of the mitochondrial PT in cytochrome *c* release and apoptosis induced by photodynamic therapy (PDT). PDT is a treatment for cancer and other abnormal tissue that employs a photosensitizer and visible light to produce singlet oxygen and other reactive oxygen species (Weishaupt et al, 1976), which cause an oxidative stress in cells and membrane damage (Moan and Berg, 1992) and eventually leads to cell death and tumour ablation (reviewed in Dougherty, 1993; Dougherty et al, 1998). PDT with photosensitizers that localize in the mitochondria induces rapid apoptosis (Agarwal et al, 1991; Dougherty, 1993; He et al, 1994; Luo et al, 1996; Luo and Kessel, 1997; Dougherty et al, 1998; Oleinick and Evans, 1998), probably because the photochemical damage directly targets mitochondria (Kessel and Luo, 1998) to elicit the rapid release of cytochrome *c* that initiates caspase-9 activation, subsequent steps in the caspase cascade, and morphological apoptosis (Kroemer et al, 1997; Granville et al, 1998, 1999; Kessel and Luo, 1999; Varnes et al, 1999). Data in the present study show that mitochondrial depolarization caused by PDT is dose dependent, and PDT-induced cytochrome *c* release and apoptosis can occur in the absence of major loss of the $\Delta\psi_m$.

### MATERIALS AND METHODS

### Photosensitizer

The silicon phthalocyanine Pc 4[$HOSiPcOSi(CH_3)_2(CH_2)_3(CH_3)_2$] was supplied by Drs Ying-syi Li and Malcolm E Kenney of the Department of Chemistry, CWRU, and used as a 0.5 mM stock solution in dimethyl formamide. For experiments, Pc 4 was added to the medium of the cell cultures to a final concentration of 300 nM.

### Cell culture and photodynamic treatment

Mouse lymphoma L5178Y-R (LY-R) cells were cultured in Fisher's medium supplemented with 5 mM glutamine and 10% heat-inactivated horse serum in a humidified atmosphere at 37˚C with 5% $CO_2$. Pc 4 was added to the cultures growing in T-25 flasks to a final concentration in the medium of 300 nM, 16–18 h before light exposure. Cells in the original culture flasks were irradiated using a light-emitting diode (LED) array (EFOS, Mississauga, Ontario, Canada, $\lambda_{max}$ 670–675 nm) followed by incubation in the dark for various periods of time before harvest.

### Nuclear staining assay for apoptotic cells

Apoptotic cells were identified by fluorescence microscopy from their characteristic nuclear features of chromatin condensation and fragmentation after staining the cells with 1–5 μg $ml^{-1}$ Hoechst 33342. At least 200 cells were counted from each sample, and the yield of apoptotic cells was expressed as the percentage of the total population.

### Fluorescence immunocytochemistry

Cells were washed in sucrose buffer (0.25 M sucrose, 10 mM Tris-HCl, 3 mM $MgCl_2$, pH 7.4) and fixed in methanol at –20˚C for 10 min. After rinsing twice with PBS, the cells were allowed to attach to gelatin-coated coverslips, which were then air-dried. The coverslips containing attached cells were incubated in IFA buffer (PBS containing 1% bovine serum albumin, 0.1% Tween 20) for 10 min and then in IFA containing mouse anti-cytochrome *c* antibody (1:100 dilution, clone 6H2.B4, PharMingen) for 1 h to overnight at 4˚C. After rinsing with IFA buffer to remove excess unbound antibody, the coverslips were incubated for at least 1 h at 4˚C in IFA containing the second antibody, which was anti-mouse IgG conjugated to Texas red (1:100 dilution, Vector Laboratories). Following thorough rinsing, the samples were stained with 0.5 μg/ml Hoechst 33342 and examined with a Leitz fluorescence microscope or a Zeiss 410 confocal microscope.

### Measurement of mitochondrial membrane potential

Changes in $\Delta\psi_m$ were monitored by the uptake of JC-1 or rhodamine-123 (Rh-123). JC-1 (5,5′,6,6′-tetrachloro-1,1′,3,3′ -tetraethylbenzimidazolylcarbocyanine iodide) was supplied by Molecular Probes and dissolved in DMSO to produce a 1 mg $ml^{-1}$ stock solution. Rh-123 was supplied by Eastman Kodak and dissolved in DMSO to produce a 1 mM stock solution. Cells were incubated at 37˚C for consecutive 15-min periods starting immediately after light exposure until 45–60 min after PDT in culture medium containing 10 μg $ml^{-1}$ JC-1 or 1 μM Rh-123. Samples labelled with JC-1 were either unwashed or washed once with HBSS before being analysed with an EPICS ESP flow cytometer (Coulter Corp) in the Flow Cytometry Facility of the Case Western Reserve University/Ireland Comprehensive Cancer Center with settings of FL1 at 530 nm and FL2 at 585 nm. A portion of the sample was also stained with Hoechst 33342 and examined with a fluorescence microscope. JC-1 is a lipophilic cationic dye which accumulates and forms aggregates in normal mitochondria with a high negative membrane potential, in which condition it emits a red-orange fluorescence, detected at 585 nm, whereas in mitochondria with low membrane potential it forms monomers in the cytosol that emit a green fluorescence, detected at 530 nm (Cossarizza et al, 1993).

For fluorescence microscopy, labelled samples were washed once with PBS and examined for red-orange fluorescence (580 nm). Despite some variation in the level of Rh123 or JC-1 fluorescence intensity among control cells, all cells displayed bright perinuclear and punctate fluorescence, an indication of the accumulation of the dye in mitochondria. In contrast, cells pre-treated with the uncoupler of oxidative phosphorylation, CCCP (carbonyl cyanide *m*-chlorophenylhydrazone), which abolishes the mitochondrial membrane potential, prior to dye labelling displayed very weak fluorescence with little or no evidence of a bright punctate staining pattern. This indicates that mitochondrial depolarization can be readily demonstrated by these dyes in LY-R cells. In the determination of the percentage of cells that had lost $\Delta\Psi_m$ following PDT, only cells with very low fluorescence were scored. At least 200 cells were scored for each sample.

### Measurement of caspase activity

Samples were prepared and assayed for caspase-3-like activity as described (Varnes et al, 1999). Briefly, samples containing 50 μg protein was incubated in 60 μl of reaction buffer (25 mM HEPES, 10% sucrose, 0.1% Chaps, 1 mM EGTA, 1 mM EDTA, 5 mM dithiothreitol, 1 mM phenylmethylsulphonyl fluoride, 100 μM

 *© 2001 Cancer Research Campaign*

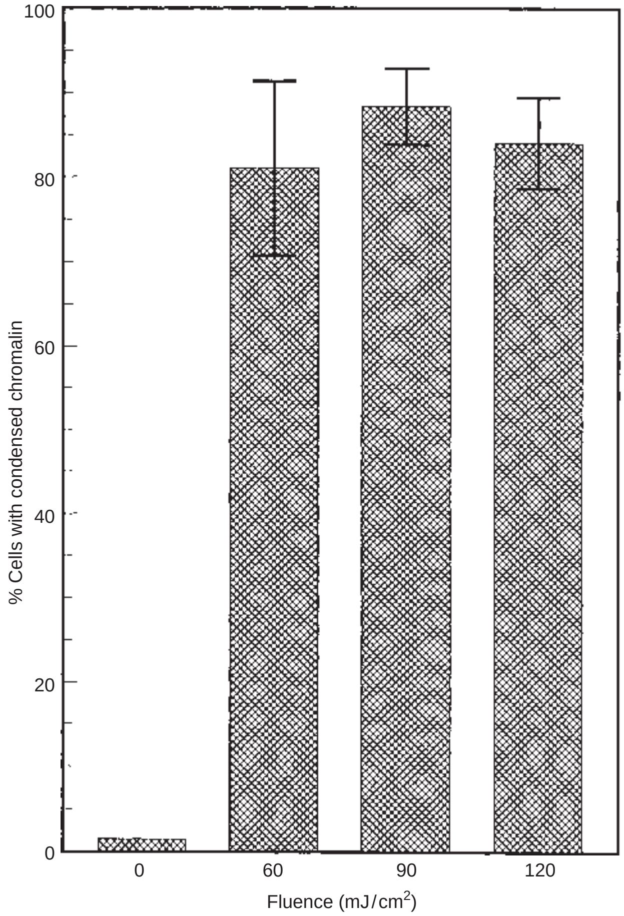

**Figure 1** Levels of apoptosis induced by various doses of PDT. LY-R cells were exposed to 300 nM Pc 4 and 0, 60, 90 or 120 mJ/cm² of red light delivered by an LED array. One hour after treatment, cells were collected and stained with Hoechst 33342 and examined by fluorescence microscopy. Apoptotic cells were identified by condensation and fragmentation of their nuclei. At least 200 cells were scored for each sample

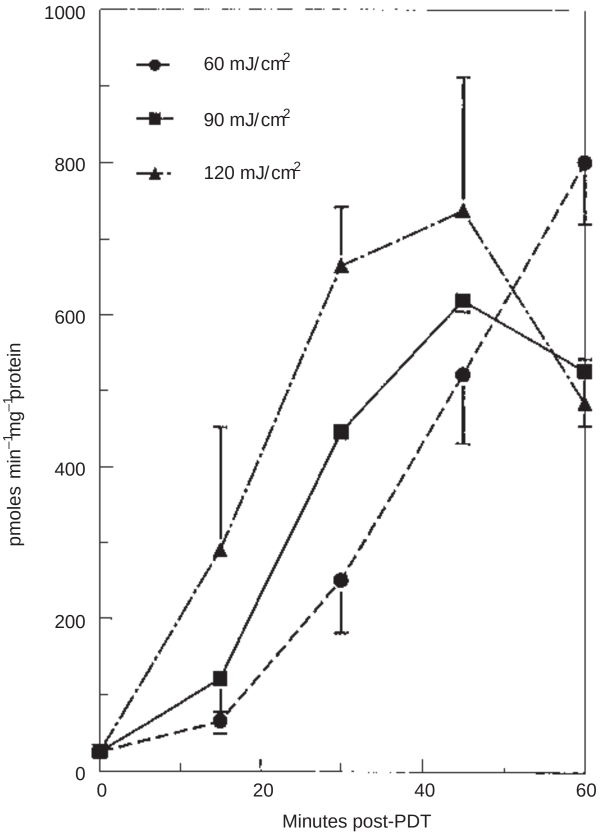

**Figure 2** Elevation of caspase-3-like protease activity in LY-R cells after various doses of PDT. LY-R cells were treated as for Figure 1 and at the times indicated, cells were collected and lysed. 50 μg of lysate protein were incubated with DEVD.AMC at 37°C for 1 h. The fluorescence intensity of released AMC was measured. Data represent the mean ± SD of at least 3 independent experiments pepstatin, 100 μM leupeptin, pH 7.4) containing 50 μM DEVD-AMC (BIOMOL) for 1 h at 37°C. The released fluorescence was measured in a Perkin-Elmer LS50 fluorometer ($\lambda_{ex}$ 380 nm; $\lambda_{em}$ 460 nm).

### RESULTS

We have previously demonstrated that the extent and timing of induction of apoptosis by Pc 4-PDT in mouse lymphoma LY-R cells was dose dependent (He et al, 1998). In order to test a possible correlation between the loss of $\Delta\Psi_m$ and cytochrome *c* release and apoptosis, 3 doses of PDT were chosen for this study; LY-R cells were treated with 300 nM Pc 4 and irradiated with either 60, 90 or 120 mJ/cm² red light. As determined by clonogenic assay, PDT with the lowest dose results in killing of 99% of the cells (Chiu et al, 2001). Figure 1 shows that all 3 PDT doses caused greater than 80% of the cells to undergo nuclear apoptosis within 1 h. An even higher incidence of apoptotic figures (92.3 ± 0.6%) was observed 2 h after these 3 doses of Pc 4-PDT, and extensive degradation of DNA to oligonucleosome-size fragments was detected at this time (not shown). Since caspase-3 is the key 'executioner' of apoptosis and our previous studies have shown its activity elevated markedly after PDT with Pc 4 in LY-R cells (He et al, 1998; Varnes et al, 1999), the activation of this caspase was investigated as a function of the same PDT doses. As shown in Figure 2, DEVDase activity rose rapidly following each dose of PDT, reaching a maximum level, 20–30-fold higher than in untreated cells, by 30–60 min after PDT. The rate of activation of caspase-3 was PDT dose-dependent, however, with the most and least rapid activation of the caspase occurring in response to the highest and lowest dose of PDT, respectively.

The effect of PDT on the loss of mitochondrial membrane potential ($\Delta\Psi_m$) was monitored by the uptake of JC-1, a cationic, lipophilic fluorescent dye that accumulates in normal mitochondria with a negative $\Delta\Psi_m$, resulting in an intramitochondrial concentration that is 2–3 logs higher than in the cytosol. Under this condition, JC-1 forms aggregates that emit red fluorescence. In contrast, disruption of $\Delta\Psi_m$ results in a much reduced uptake of JC-1. This dye thus provides a very sensitive method to measure changes in $\Delta\Psi_m$. To monitor the effect of the chosen doses of Pc 4-PDT on mitochondrial $\Delta\Psi_m$, cells were allowed to take up JC-1 during consecutive 15-min periods from immediately following PDT until 60 min later at which time the treated cells were in

© 2001 Cancer Research Campaign 

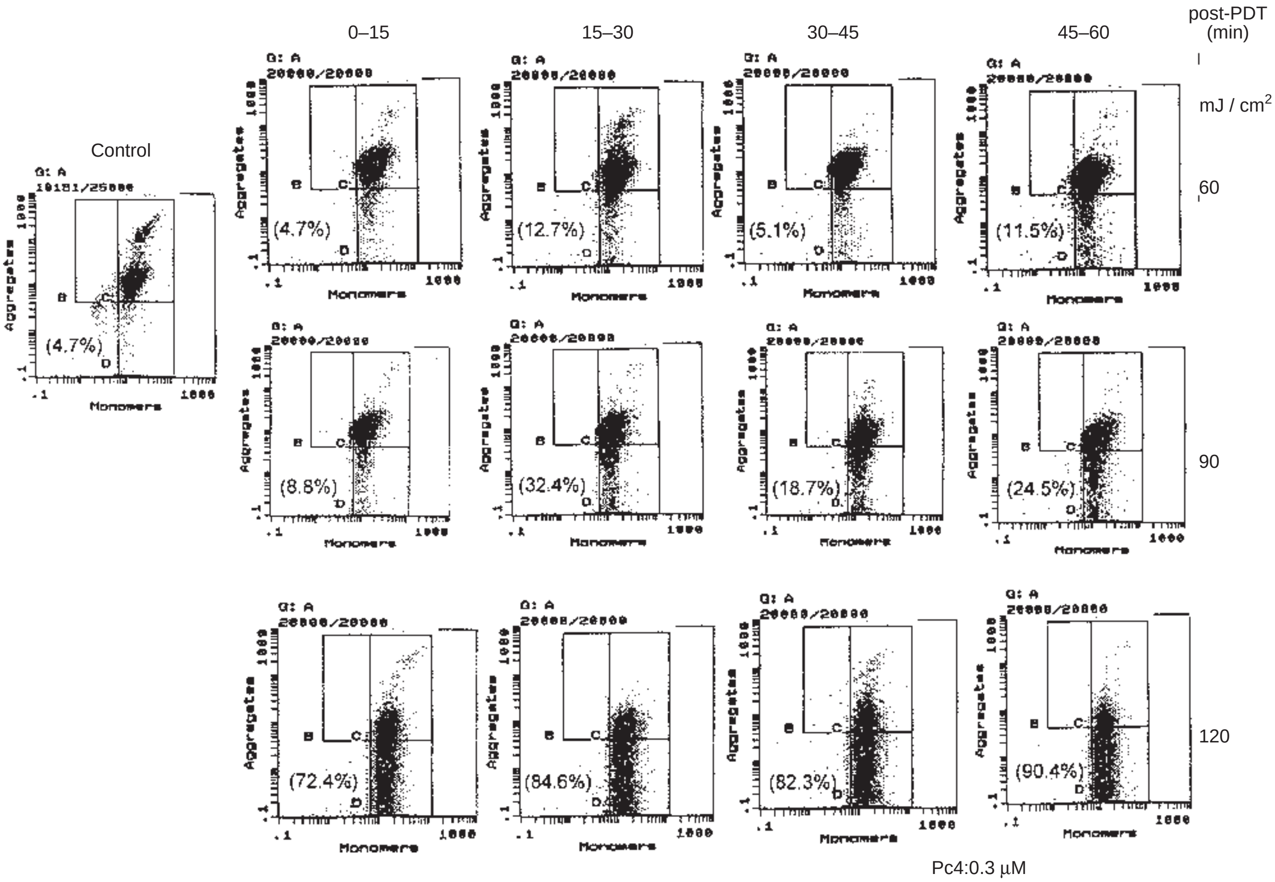

**Figure 3** Time-course of changes in mitochondrial membrane potential after PDT, as determined by the uptake of JC-1. LY-R cells were treated as described for Figure 1. At consecutive 15-min intervals thereafter, cells were collected and incubated in medium containing JC-1 for 15 min. The labelled cells were then analysed by flow cytometry to measure the uptake of JC-1, an indicator of mitochondrial membrane potential ($\Delta\psi_m$). In the histograms of cells stained with JC-1, the labels 'Aggregates' for the ordinate and 'Monomers' for the abscissa are derived from the fluorescence readings in the 2 channels, FL2 (585 nm) and FL1 (530 nm), of the flow cytometer, respectively. Thus cells with high red fluorescence (high $\Delta\psi_m$) are found in the upper right quadrant (box C); those with low $\Delta\psi_m$ are in the lower right quadrant (box D). The percentage in parentheses in the lower left of each profile represents the percentage of the total cells with low fluorescence intensity (box D of each histogram)

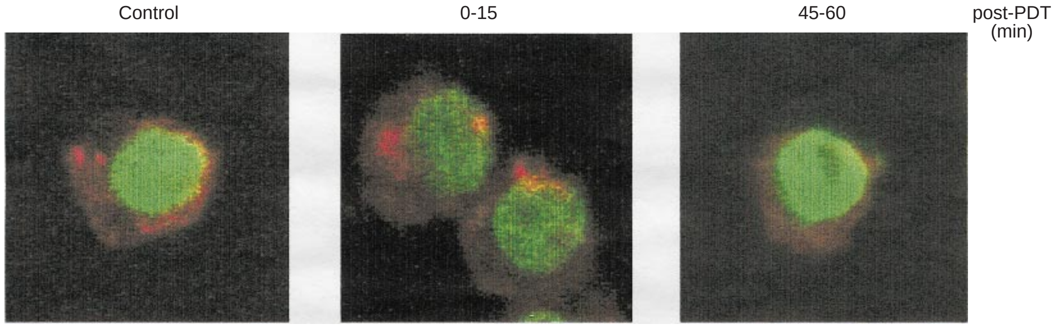

**Figure 4** Fluorescence micrographs of LY-R cells stained with JC-1 (red) and Hoechst 33342 (green). LY-R cells receiving 300 nM Pc 4 and either 0 or 90 mJ/cm² red light were labelled with JC-1 as for Figure 3, stained with Hoechst 33342, and visualized by confocal microscopy nuclear apoptosis. The labelled cells were analysed either by flow cytometry (Figure 3) or by fluorescence microscopy (Figure 4).

The flow cytometric analysis of JC-1 uptake following each of the 3 doses of PDT shows clearly that the observed effect of PDT

 *© 2001 Cancer Research Campaign*

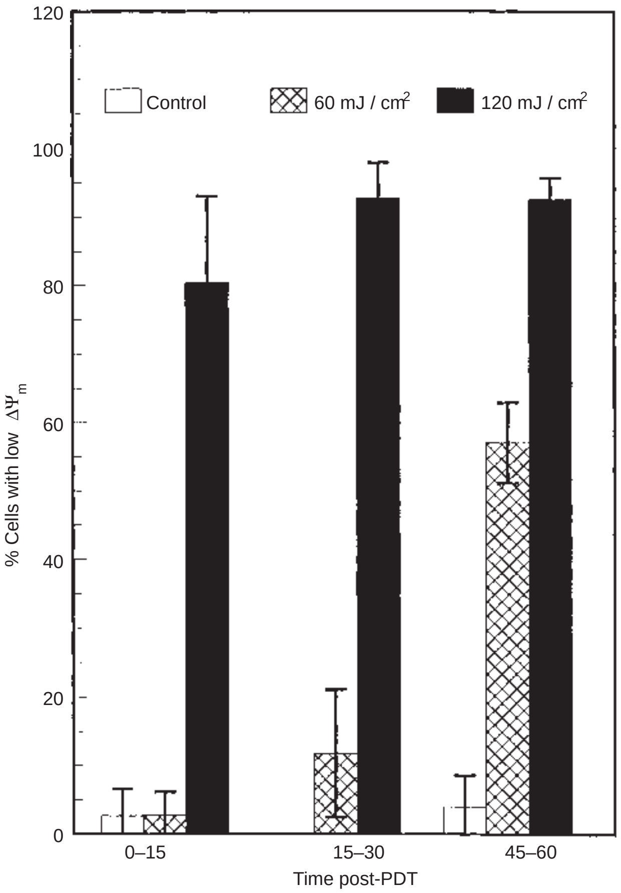

**Figure 5** Effect of PDT on the mitochondrial membrane potential of LY-R cells, as measured by the uptake of Rh-123. LY-R cells treated with 300 nM Pc 4 were exposed to 0, 60 or 120 mJ/cm² red light and allowed to take up Rh-123, as described in Materials and Methods. The data represent the mean ± SD from 3 experiments on $\Delta\Psi_m$ is dependent on both the dose and the post-treatment time (Figure 3). The highest dose of PDT (300 nM Pc 4 + 120 mJ/cm²) caused a marked reduction of JC-1 uptake immediately after PDT in >70% of the cells. Thereafter, the fraction of cells with low $\Delta\Psi_m$ increased gradually to about 90% during the 45–60 min time period. In contrast, there was little or no immediate change in $\Delta\Psi_m$ in cells receiving either of the lower doses of Pc 4-PDT. However, at later times, up to 13 and 34% of the cells revealed low $\Delta\Psi_m$ after exposure to 60 and 90 mJ/cm², respectively. A portion of the JC-1 labelled cells was also stained with Hoechst 33342 and examined by confocal microscopy. An example of cells treated with 300 nM Pc 4 and 90 mJ/cm² of red light is displayed in Figure 4. In control untreated cells, JC-1 fluorescence was found to have a perinuclear distribution and a punctuate pattern, indicating its location in mitochondria (Ankarcrona et al, 1996). A slight reduction in JC-1 fluorescence was observed in cells 15 min after receiving 90 mJ/cm². However, in these cells the JC-1 fluorescence pattern was found clustered at 1 or 2 locations within the cytoplasm, in contrast to the more or less randomly distributed punctuate fluorescence found for untreated cells. By 60 min after PDT, when the majority of cells displayed condensed and fragmented chromatin, JC-1 fluorescence was reduced in some of the treated cells.

To confirm that the change in mitochondrial uptake of a cationic dye after PDT is not unique to JC-1, another mitochondrial dye was tested. Rh-123, like JC-1, is a cationic dye that accumulates into energized mitochondria in response to their negative membrane potential; this dye has been widely used as a measure of mitochondrial polarization (Johnson et al, 1981; Emaus et al, 1986). Following exposure of LY-R cells to PDT with 60 or 120 mJ/cm², cells were labelled with Rh-123 in a manner similar to that for JC-1, and cells with low $\Delta\Psi_m$ (low Rh-123 uptake) were observed with a fluorescence microscope and counted. The results shown in Figure 5 indicate a similar effect on $\Delta\Psi_m$ as that found using JC-1. For PDT with 60 mJ/cm², there was no significant

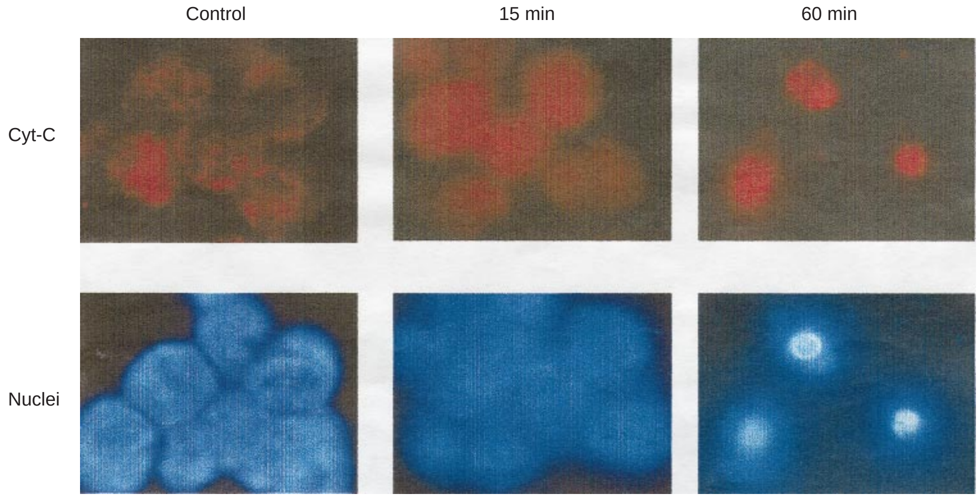

**Figure 6** Cytochrome *c* redistribution visualized by fluorescence microscopy. Immunolocalization of cytochrome *c* (red) and staining of nuclei with Hoechst 33342 (blue) are shown in untreated cells (left images), and in cells collected at 15 min (middle images) or 60 min (right images) after receiving PDT (300 nM Pc 4 + 60 mJ/cm²). Cells were fixed and immunostained with anti-cytochrome *c* (clone 6H2.B4)

© 2001 Cancer Research Campaign 

increase in the number of cells with low $\Delta\Psi_m$ at early times after treatment, although nearly 60% of the cells revealed a low $\Delta\Psi_m$ by 45–60 min. For PDT with 120 mJ/cm$^2$, 80% of the cells had lost $\Delta\Psi_m$ at the earliest time period.

Previously, we and others showed that PDT causes cytochrome *c* to be released from the mitochondria into the cytosol prior to caspase activation (Granville et al, 1998, 1999; Kessel and Luo, 1999; Varnes et al, 1999). However, in those studies, the presence of cytochrome *c* in the cytosol was determined by Western blot analysis following cell homogenization and differential centrifugation. Because of the potential for artifacts due to the physical stress on organelles during cell fractionation, we employed an immunocytochemical method to monitor the redistribution of cytochrome *c* from mitochondria to cytosol. Since we used mouse anti-cytochrome *c* for this assay, it was important to test that the LY-R mouse lymphoma cells do not produce sufficient immunoglobulin IgG to react with the second antibody and interfere with the assay. We found that omission of the first antibody produced a very clean background in cell preparations, indicating no appreciable synthesis of IgG by these cells. In contrast, cells that had reacted with the mouse anti-cytochrome *c* antibody before reaction with the second antibody revealed a bright perinuclear punctate fluorescence pattern (Figure 6). The perinuclear location and punctate pattern of cytochrome *c* staining is consistent with previous reports (Bossy-Wetzel et al, 1998; Rosse et al, 1998; Srinivasan et al, 1998), indicative of the mitochondrial location of cytochrome *c*.

Examples of control and PDT-treated (300 nM Pc 4 and 60 mJ/cm$^2$ red light) LY-R cells examined for cytochrome *c* distribution and nuclear morphology are displayed in Figure 6. The staining of untreated cells for cytochrome *c* revealed a punctate pattern, consistent with localization in the mitochondria, and nuclear morphology was normal. Treated cells maintained the punctate cytochrome *c* staining pattern until 10 min post-PDT, indicating no release of cytochrome *c* from mitochondria at this time (not shown). In contrast, by 15 min after PDT the majority of the treated cells showed a diffuse distribution of red fluorescence, indicating the release of cytochrome *c* from mitochondria. Chromatin condensation started at 45 min and fragmentation at 60 min post-PDT, and apoptotic cells continued to show the diffuse cytochrome *c* staining pattern. At later times, the staining of cytochrome *c* became very weak (not shown), probably because the protein leaked out of the cells (Heiskanen et al, 1999). The majority of the cells that received the higher dose of PDT (300 nM Pc 4 and 120 mJ/cm$^2$ red light) also released cytochrome *c* from mitochondria at 15 min (data not shown).

### DISCUSSION

The 3 doses of PDT employed in this study induced a similar high level (80–90%) of cells to undergo apoptosis by 1 h, yet the responses of the cells in terms of changes in their mitochondrial membrane potential were strikingly different. The effect of PDT on $\Delta\Psi_m$ was dose- and time-dependent: whereas the highest dose of PDT caused >70% of the cells to lose $\Delta\Psi_m$ immediately after irradiation, virtually all cells receiving lower doses of PDT showed no significant change in $\Delta\Psi_m$ immediately after PDT, although 25–60% of the cells showed dissipation of $\Delta\Psi_m$ at later times coincident with nuclear apoptosis. Thus, with the lower doses of PDT, the majority of the cells could initiate apoptosis in the absence of a measurable loss of $\Delta\Psi_m$.

According to recent studies (D Godar, personal communication), JC-1 is susceptible to oxidation by reactive oxygen species, and the resultant oxidized JC-1 may either lose fluorescence or show green instead of red fluorescence. Although PDT generates reactive oxygen species that could oxidize JC-1, our observation of an immediate loss of $\Delta\Psi_m$ measured with JC-1 following the highest dose of PDT appears unlikely to be due to a false reading of oxidized JC-1, because we were able to repeat the observation with Rh-123, which has also been demonstrated to be a specific probe for $\Delta\Psi_m$ (Johnson et al, 1981; Emaus et al, 1986). The loss of $\Delta\Psi_m$ after the highest dose of PDT is likely due to heavy photodamage to mitochondrial membranes, since mitochondria are the most sensitive target of PDT with many currently used photosensitizers (Kessel and Luo, 1998). Loss of $\Delta\Psi_m$ has been demonstrated in murine leukaemia P388 cells after PDT with the mitochondrion-localizing photosensitizers PcM (porphycene monomer) and CPO (9-capronyloxy-tetrakis (methyloxyethyl) porphycene); the response was observed even when photoirradiation was carried out at 10˚C, a temperature that would suppress most metabolic processes, suggesting that direct photodamage to mitochondrial membranes was responsible (Kessel and Luo, 1999).

Release of cytochrome *c* in the absence of dissipation of $\Delta\Psi_m$ has also been reported for HeLa cells after PDT with Verteporfin (Carthy et al, 1999). In contrast, a nearly instantaneous depolarization of mitochondria has been shown following PDT with PcM and CPO in P388 cells, as described above (Kessel and Luo, 1999), as well as following treatment of mouse fibroblasts with PDT sensitized by hypericin (Chaloupka et al, 1999). The controversy in these results could be resolved by careful attention to the delivered dose of PDT, as demonstrated in this study. However, other factors may also contibute to the variability in findings, such as differences in the responses of the cells and/or differences in the photosensitizers employed and their subcellular binding sites. In a study of the induction of cytochrome *c* release from isolated mitochondria by exogenous Bax (Pastorino et al, 1999), both the amount of cytochrome *c* released and the extent of mitochondrial swelling and depolarization were dependent on the concentration of Bax. While a low concentration (125 nM) of Bax produced no detectable mitochondrial swelling or depolarization, higher concentrations (250–1000 nM) produced persistent mitochondrial changes. Our results thus confirm the importance of including a range of doses of the apoptosis-initiating agent in any study of the relationship between membrane potential, cytochrome *c* release, and apoptosis.

Using an immunocytochemical procedure to locate cytochrome *c*, we have found that the majority of the cytochrome *c* was released from mitochondria at 15 min after PDT, regardless of the dose. The timing of cytochrome *c* release determined by this method is similar to the 10–20 min after PDT found previously by Western blot analysis (Varnes et al, 1999). Except for the highest dose of PDT (120 mJ/cm$^2$), very little increase in caspase-3 activity was observed at this time (Figure 2). The results confirm that the release of cytochrome *c* is coincident with or precedes the onset of caspase activation. Furthermore, at the time of cytochrome *c* release (15 min post-PDT) only a small fraction of the cells receiving one of the lower doses of PDT had lost $\Delta\Psi_m$. These data are in agreement with the previous conclusion (Finucane et al, 1999a, 1999b; Goldstein et al, 2000) that dissipation of $\Delta\Psi_m$ is not required for the release of cytochrome *c* from mitochondria. However, the possibility of a transient depolarization prior to cytochrome *c* release without affecting the overall mitochondrial $\Delta\Psi_m$ and JC-1 labelling cannot be ruled out.

© 2001 Cancer Research Campaign

A recent study (Goldstein et al, 2000) followed the release of Green Fluorescent Protein-tagged cytochrome *c* during apoptosis in HeLa cells induced by a variety of agents, finding that all of the cytochrome *c* is released from all mitochondria within a given cell in a short constant time period of 5 min, irrespective of the timing of the onset of release, which depended on the type and strength of the particular apoptotic stimulus. In that work, the release of cytochrome *c* differed from the loss of $\Delta\Psi_m$ in that only the latter depended on caspase activity. Even though the release of cytochrome *c* was accompanied by mitochondrial depolarization, in the presence of the pan-caspase inhibitor zVAD-fmk, cytochrome *c* was released to the cytosol in the absence of loss of $\Delta\Psi_m$. To explain their results, the authors (Goldstein et al, 2000) proposed the formation of holes, tears or pores in the mitochondrial outer membrane without an alteration of the inner membrane, which maintains $\Delta\Psi_m$. Such perforations in the outer membrane would allow cytochrome *c* and other mitochondrial proteins residing in the intermembrane space to diffuse out of the mitochondria in a relatively temperature-independent manner. Such a mechanism may explain certain responses of mitochondria of LY-R cells treated with Pc 4-PDT. We have found (Varnes et al, 1999) that the inhibition of respiration that occurs in response to Pc 4-PDT can be largely reversed by the addition of exogenous cytochrome *c* to digitonin-permeabilized LY-R cells. The results suggest that the respiratory inhibition is due to the loss of cytochrome *c* from the mitochondria rather than to extensive damage to the inner membrane and respiratory complexes. Thus, in LY-R cells exposed to lethal, but not supralethal, doses of Pc 4-PDT, the efflux of cytochrome *c* may be triggered by photodamage to the outer mitochondrial membrane.

Although the role of cytochrome *c* in caspase activation is well documented, little is known concerning the detailed mechanism for the regulation of caspase activation by cytochrome *c*. Our data show that the kinetics of caspase-3-like activation by PDT is dose dependent with higher doses of PDT inducing faster activation of the caspase. In spite of these differences in kinetics, no detectable difference was found in the timing or the extent of cytochrome *c* release after the PDT doses studied. These observations suggest the existence of an additional level of regulation of caspase activation after cytochrome *c* release.

### ACKNOWLEDGEMENTS

The authors thank Dr Minh Lam for assistance in scanning confocal microscopy and Liang-yan Xue for the preparation of figures. This research was supported by USPHS Grants P01 CA48735 and P30 CA43703 from the National Cancer Institute, DHHS.

### REFERENCES

Agarwal ML, Clay ME, Harvey EJ, Evans HH, Antunez AR and Oleinick NL (1991) Photodynamic therapy induces rapid cell death by apoptosis in L5178Y mouse lymphoma cells. *Cancer Res* **51**: 5993–5996

Ankarcrona M, Dypbukt JM, Orrenius S and Nicotera P (1996) Calcineurin and mitochondrial function in glutamate-induced neuronal cell death. *FEBS Lett* **394**: 321–324

Bossy-Wetzel E, Newmeyer DD and Green DR (1998) Mitochondrial cytochrome *c* release in apoptosis occurs upstream of DEVD-specific caspase activation and independently of mitochondrial transmembrane depolarization. *EMBO J* **17**: 37–49

Carthy CM, Granville DJ, Jiang H, Levy J, Rudin CM, Thompson CB, McManus BM and Hunt DWC (1999) Early release of mitochondrial cytochrome c and expression of mitochondrial epitope 7A6 with a porphyrin-derived photosensitizer: Bcl-2 and Bcl-xL overexpression do not prevent early mitochondrial events but still depress caspase activity. *Lab Invest* **79**: 953–965.

Chaloupka R, Petit PX, Israel N and Sureau F (1999) Overexpression of Bcl-2 does not protect cells from hypericin photo-induced mitochondrial membrane depolarization, but delays subsequent events in the apoptotic pathway. *FEBS Lett* **462**: 295–301

Chiu SM, Evans HH, Lam M, Nieminen AL and Oleinick NL (2001) Phthalocyanine 4 photodynamic therapy-induced apoptosis of mouse L51787 - R cells results from a delayed but extensive release of cytochrome C from mitochondria. *Cancer Le H* (in press)

Cortese JD, and Hackenbrock CR (1993) Motional dynamics of functional cytochrome c delivered by low pH fusion into the intermembrane space of intact mitochondria. *Biochim Biophys Acta* **1142**: 194–202

Cossarizza A, Baccarani Contri M, Kalashnikova G and Fransceschi C (1993) A new method for the cytofluorimetric analysis of mitochondrial membrane potential using the J-aggregate forming lipophilic cation 5, 5′, 6,6′-tetrachloro-1, 1′, 3,3′ -tetraethylbenzimidazolylcarbocyanine iodide (JC-1). *Biochem Biophys Res Commun* **197**: 40–45

Dougherty TJ (1993) Photodynamic therapy. *Photochem Photobiol* **58**: 895–900

Dougherty TJ, Gomer CJ, Henderson BW, Jori G, Kessel D, Korbelik M, Moan J and Peng Q (1998) Photodynamic Therapy: Review. *J Natl Cancer Inst* **90**: 889–902

Emaus RK, Grunwald R and Lemasters JJ (1986) Rhodamine 123 as a probe of transmembrane potential in isolated rat-liver mitochondria: special and metabolic properties. *Biochim Biophys Acta* **850**: 436–448

Enari M, Sahahira H, Yokoyama H, Okawa K, Iwamatsu A and Nagata S (1998) A caspase-activated DNase that degrades DNA during apoptosis, and its inhibitor ICAD. *Nature* **391**: 43–50

Eskes R, Antonsson B, Osen-Sand A, Montessuit S, Richter C, Sadoul R, Mazzei G, Nichols A and Martinou J-C (1998) Bax-induced cytochrome c release from mitochondria is independent of the permeability transition pore but highly dependent on $M_g^{2+}$ ions. *J Cell Biol* **143**: 216–224

Finucane DM, Bossy-Wetzel E, Waterhouse NJ, Cotter TG and Green DR (1999a) Bax-induced caspase activation and apoptosis via cytochrome c release from mitochondria is inhibited by Bcl-xL. *J Biol Chem* **274**: 2225–2233

Finucane DM, Waterhouse NJ, Amarante-Mendes GP, Cotter TG and Green DR (1999b) Collapse of the inner mitochondrial transmembrane potential is not required for apoptosis of HL60 cells. *Exp Cell Res* **251**: 166–174

Goldstein JC, Waterhouse NJ, Julin P, Evan GI and Green DR (2000) The coordinate release of cytochrome c during apoptosis is rapid, complete and kinetically invariant. *Nature Cell Biol* **2**: 156–162

Granville DJ, Carthy CM, Jiang H, Shore GC, McManus BM and Hunt DW (1998) Rapid cytochrome c release, activation of caspases 3, 6, 7 and 8 followed by Bap31 cleavage in HeLa cells treated with photodynamic therapy. *FEBS Lett* **437**: 5–10

Granville DJ, Jiang H, An MT, Levy JG, McManus BM and Hunt DWC (1999) Bcl-2 overexpression blocks caspase activation and downstream apoptotic events instigated by photodynamic therapy. *Br J Cancer* **79**: 95–100

Green DR and Reed JC (1998) Mitochondria and apoptosis. *Science* **281**: 1309–1312

Gross A, McDonnell JM and Korsmeyer SJ (1999) Bcl-2 family members and the mitochondria in apoptosis. *Genes & Develop* **13**: 1899–1911

He XY, Sikes RA, Thomsen S, Chung LWK and Jacques SL (1994) Photodynamic therapy with Photofrin II induces programmed cell death in carcinoma cell lines. *Photochem Photobiol* **59**: 468–473

He J, Whitacre CM, Xue L-y, Berger NA and Oleinick NL (1998) Protease activation and cleavage of Poly (ADP-ribose) polymerase: An integral part of apoptosis in response to photodynamic treatment. *Cancer Res* **58**: 940–946

Heiskanen KM, Bhat MB, Wang H-W, Ma J, and Nieminen A-L (1999) Mitochondrial depolarization accompanies cytochrome c release during apoptosis in PC6 cells. *J Biol Chem* **274**: 5654–5658

Johnson LV, Walsh ML, Bockus BJ and Chen LB (1981) Monitoring of relative mitochondrial membrane potential in living cells by fluorescence microscopy. *J Cell Biol* **88**: 526–535

Kessel D and Luo Y (1998) Mitochondrial photodamage and PDT-induced apoptosis. *J Photochem Photobiol B Biol* **42**: 89–95

Kessel D and Luo Y (1999) Photodynamic therapy: a mitochondrial inducer of apoptosis. *Cell Death Differ* **6**: 28–35

Kluck RM, Bossy-Wetzel E, Green DR and Newmeyer DD (1997) The release of cytochrome c from mitochondria: a primary site for Bcl-2 regulation of apoptosis. *Science* **275**: 1132–1136

© 2001 Cancer Research Campaign 

Kroemer G, Zamzami N and Susin SA (1997) Mitochondrial control of apoptosis. *Immunol Today* **18**: 44–51
Lemasters JJ, Nieminen A-L, Qian T, Trost LC, Elmore SP, Nishimura Y, Crowe RA, Cascio WE, Bradham CA, Brenner DA and Herman B (1998) The mitochondrial permeability transition in cell death: a common mechanism in necrosis, apoptosis and autophagy. *Biochim Biophys Acta* **1366**: 177–196
Liu X, Kim CN, Yang J, Jemmerson R and Wang X (1996) Induction of apoptotic program in cell-free extracts: requirement for dATP and cytochrome c. *Cell* **86**: 147–157
Liu X, Zou H, Slaughter C and Wang X (1997) DFF, a heterodimeric protein that functions downstream of caspase-3 to trigger DNA fragmentation during apoptosis. *Cell* **89**: 175–184
Luo Y and Kessel D (1997) Initiation of apoptosis *versus* necrosis by photodynamic therapy with chloroaluminum phthalocyanine. *Photochem Photobiol* **66**: 479–483
Luo Y, Chang CK and Kessel D (1996) Rapid initiation of apoptosis by photodynamic therapy. *Photochem Photobiol* **63**: 528–534
Martinou J-C, Desagher S and Antonsson B (2000) Cytochrome c release from mitochondria: all or nothing. *Nature Cell Biol* **2**: E41–E43
Moan J and Berg K (1992) Photochemotherapy of cancer: experimental research. *Photochem Photobiol* **55**: 931–948
Oleinick NL and Evans HH (1998) The photobiology of photodynamic therapy: Cellular targets and mechanisms. *Radiat Res* **150**: S146–S156
Pastorino JG, Tafani M, Rothman RJ, Marcineviciute A, Hoek JB and Farber JL (1999) Functional consequences of the sustained or transient activation by Bax of the mitochondrial permeability transition pore. *J Biol Chem* **274**: 31734–31739
Reed JC, Jurgensmeier JM and Matsuyama S (1998) Bcl-2 family proteins and mitochondria. *Biochim Biophys Acta* **1366**: 127–137
Rosse T, Olivier R, Monney L, Rager M, Conus S, Fellay I, Jansen B and Borner C (1998) Bcl-2 prolongs cell survival after Bax-induced release of cytochrome c. *Nature* **391**: 496–499
Srinivasan A, Li F, Wong A, Kodandapani L, Smidt RJr, Krebs JF, Fritz LC, Wu JC and Tomaselli KJ (1998) Bcl-xl functions downstream of caspase-8 to inhibit Fas-and tumor necrosis factor receptor 1-induced apoptosis of MCF7 breast carcinoma cells. *J Biol Chem* **273**: 4523–4529
Susin SA, Zamzami N and Kroemer G (1998) Mitochondria as regulators of apoptosis: doubt no more. *Biochim Biophys Acta* **1366**: 151–165
Susin SA, Lorenzo HK, Zamzami N, Marzo I, Snow BE, Brothers GM, Mangion J, Jacotot E, Costantini P, Loeffler M, Larochette N, Goodlett DR, Aebersold R, Siderovski DP, Penninger JM and Kroemer G (1999) Molecular characterization of mitochondrial apoptosis-inducing factor. *Nature* **397**: 441–446
Vander Heiden MG, Chandel NS, Williamson EK, Schumacker PT and Thompson CB (1997) Bcl-xl regulates the membrane potential and volume homeostasis of mitochondria. *Cell* **91**: 627–637
Varnes ME, Chiu SM, Xue LY and Oleinick NL (1999) Photodynamic therapy-induced apoptosis in lymphoma cells: translocation of cytochrome c causes inhibition of respiration as well as caspase activation. *Biochem Biophys Res Commun* **255**: 673–679
Weishaupt KR, Gomer CJ and Dougherty TJ (1976) Identification of singlet oxygen as the cytotoxic agent in photoinactivation of a murine tumor. *Cancer Res* **36**: 2326–2329
Yang J, Liu X, Bhalla K, Kim CN, Ibrado AM, Cai J, Peng TI, Jones DP and Wang X (1997) Prevention of apoptosis by Bcl-2: release of cytochrome c from mitochondria blocked. *Science* **275**: 1129–1132
Zoratti M and Szabo I (1995) The mitochondrial permeability transition. *Biochim Biophys Acta* **1241**: 139–176

 *© 2001 Cancer Research Campaign*
